# Combination of metagenomic next-generation sequencing and morphology for identifying *Coccidioides immitis*: a case report

**DOI:** 10.3389/fmed.2024.1500014

**Published:** 2025-01-22

**Authors:** Hong Liu, Kaixin Zhou, Chaoran Zhong, Ming Guan

**Affiliations:** Department of Laboratory Medicine, Huashan Hospital, Fudan University, Shanghai, China

**Keywords:** *Coccidioides immitis*, coccidioidomycosis, metagenomic next-generation sequencing (mNGS), diagnosis strategy, meningitis

## Abstract

Coccidioidomycosis is a systemic infection caused by the dimorphic fungus *Coccidioides* spp., endemic to the Southwestern United States and Central and South America. In this article, we report a case of *Coccidioides immitis*-induced meningitis in a 27-year-old man who was experiencing recurrent fever and headache. His cerebrospinal fluid (CSF) biochemical markers indicated an infection in the central nervous system. However, repeated routine cultures of the CSF for bacterial detection were all negative. Only metagenomic next-generation sequencing (mNGS) detected low reads of *C. immitis*. To verify the mNGS results, the Clinical Microbiology Laboratory in Huashan Hospital optimized its culture conditions. Ultimately, 12 days after sampling, the fungal bottle containing the cerebrospinal fluid tested positive. Furthermore, the diagnosis of *C. immitis* was then confirmed by smear staining combined with morphological characteristics of the colony, which provided an important etiological basis for clinical diagnosis and treatment. As coccidioidomycosis is a rare disease in China, its pathogen-specific diagnostic methods are limited. In this case, we combined two universal methods, mNGS and traditional morphological observation, to confirm the diagnosis. This combined strategy is critical for quick and accurate diagnosis.

## Introduction

*Coccidioides* is a type of dimorphic fungus that includes *C. immitis* and *C. posadasii*. Epidemiological data have shown that *Coccidioides* is mainly prevalent in central and southern California, the low-lying desert regions of Arizona, southeastern New Mexico, western Texas, the southwestern United States, Mexico, Central America, and South America, with strong geographical distribution patterns (1). Inhalation of arthroconidia into the lungs is the primary exposure factor. Coccidioidomycosis commonly manifests as an asymptomatic infection or a mild respiratory infection in humans and other vertebrate hosts. Occasionally, approximately 4.7% of coccidioidomycosis cases are reported to spread from the lungs to other organs, such as the skin, bone, spine, and meninges. This condition is known as disseminated coccidioidomycosis (2), which usually develops rapidly. Failure to diagnose and treat this condition in time can result in poor prognosis and even death (2, 3). Therefore, early and accurate diagnosis is crucial for the treatment of disseminated coccidioidomycosis.

Coccidioidomycosis is rarely reported in China. From 1958 to 2021, only 47 cases were reported, and the majority of these cases have a history of travel to endemic areas (4, 5). As it is rare in China, the majority of clinical laboratories do not carry out *Coccidioides-*specific routine detections. In addition, its clinical signs and imaging presentations are non-specific, making it easy to misdiagnosis as a tumor or another disease (1, 6, 7). Therefore, diagnosing coccidioidomycosis is difficult in non-endemic areas (8).

This article reports on a patient with unexplained recurrent fever and headache who was transferred to our hospital after receiving ineffective treatment at a local hospital. The cerebrospinal fluid (CSF) metagenomic next-generation sequencing (mNGS) analysis revealed low reads of *C. immitis-*specific gene sequences, but the repeated CSF bacterial cultures showed no pathogens. To confirm the diagnosis, the Clinical Microbiology Laboratory in Huashan Hospital extended the CSF culture duration, based on the growth characteristics of *Coccidioides* reported in the literature (9, 10). Ultimately, 12 days after sampling, the fungal bottle containing the cerebrospinal fluid tested positive. Furthermore, the diagnosis of *C. immitis* was then confirmed by smear staining and the morphological characteristics of the colony, providing an important etiological basis for clinical diagnosis and treatment. This article will describe and discuss this case along with relevant insights.

## Case presentation

A 27-year-old male patient was admitted to the emergency department of Huashan Hospital due to a headache and fever on 11 August 2022. Around 2 months (25 June) earlier, he had been diagnosed with community-acquired pneumonia with symptoms including cough and irregular fever (up to 39.0°C) at the local hospital (Yangzhou, Jiangsu). After antibacterial treatment, the symptoms improved significantly. However, half a month earlier (31 July), the patient developed a persistent headache and fever, and the symptoms progressively worsened. For further diagnosis, he visited our hospital.

**Figure 1:**
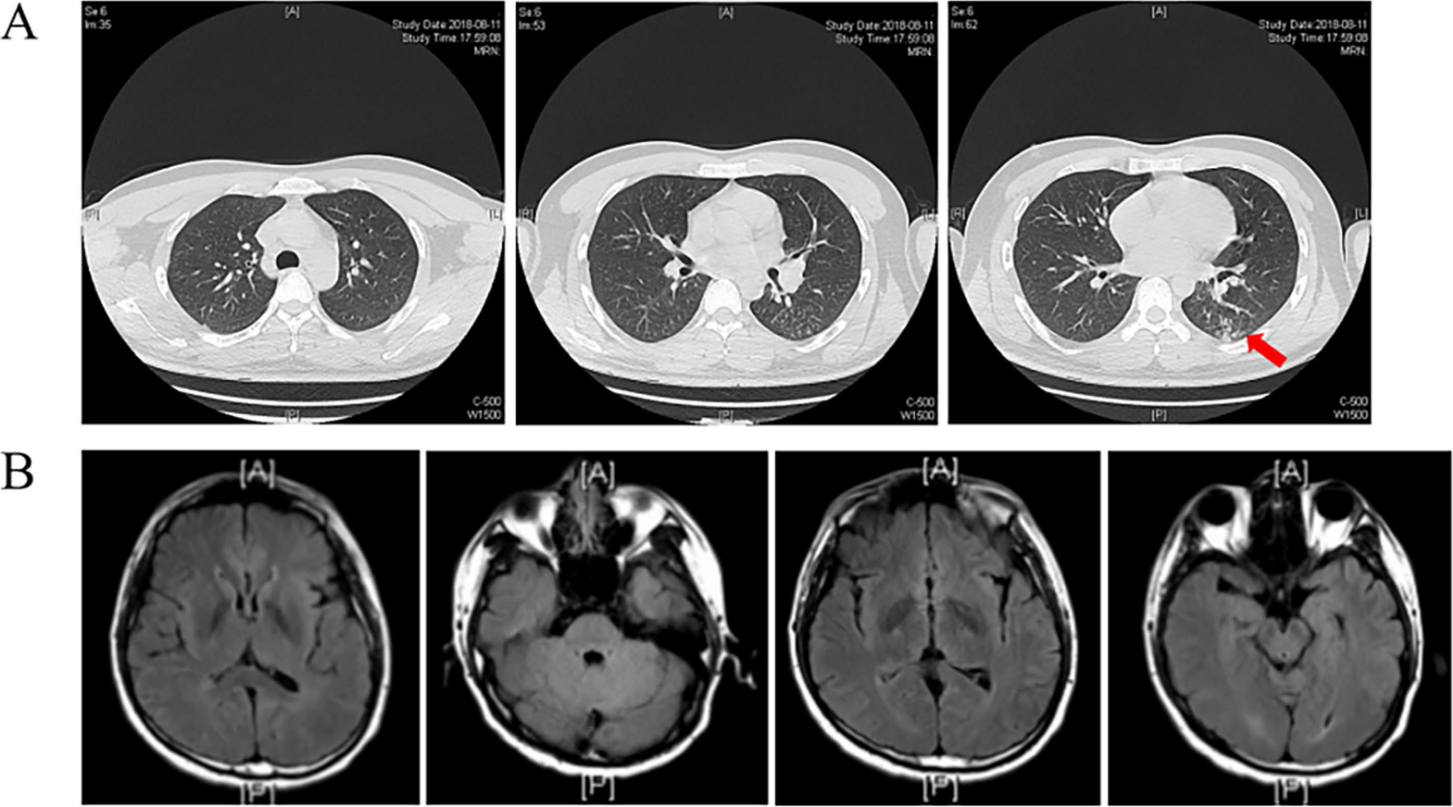
**(A)** Chest CT scan shows increased pulmonary markings in both lungs, with scattered patchy shadows in the left lower lobe (Arrow indicated). The trachea and main bronchi were patent. No enlarged lymph nodes were seen in the mediastinum or hilum; **(B)** Enhanced CT of the head shows no apparent abnormalities.

**Figure 2:**
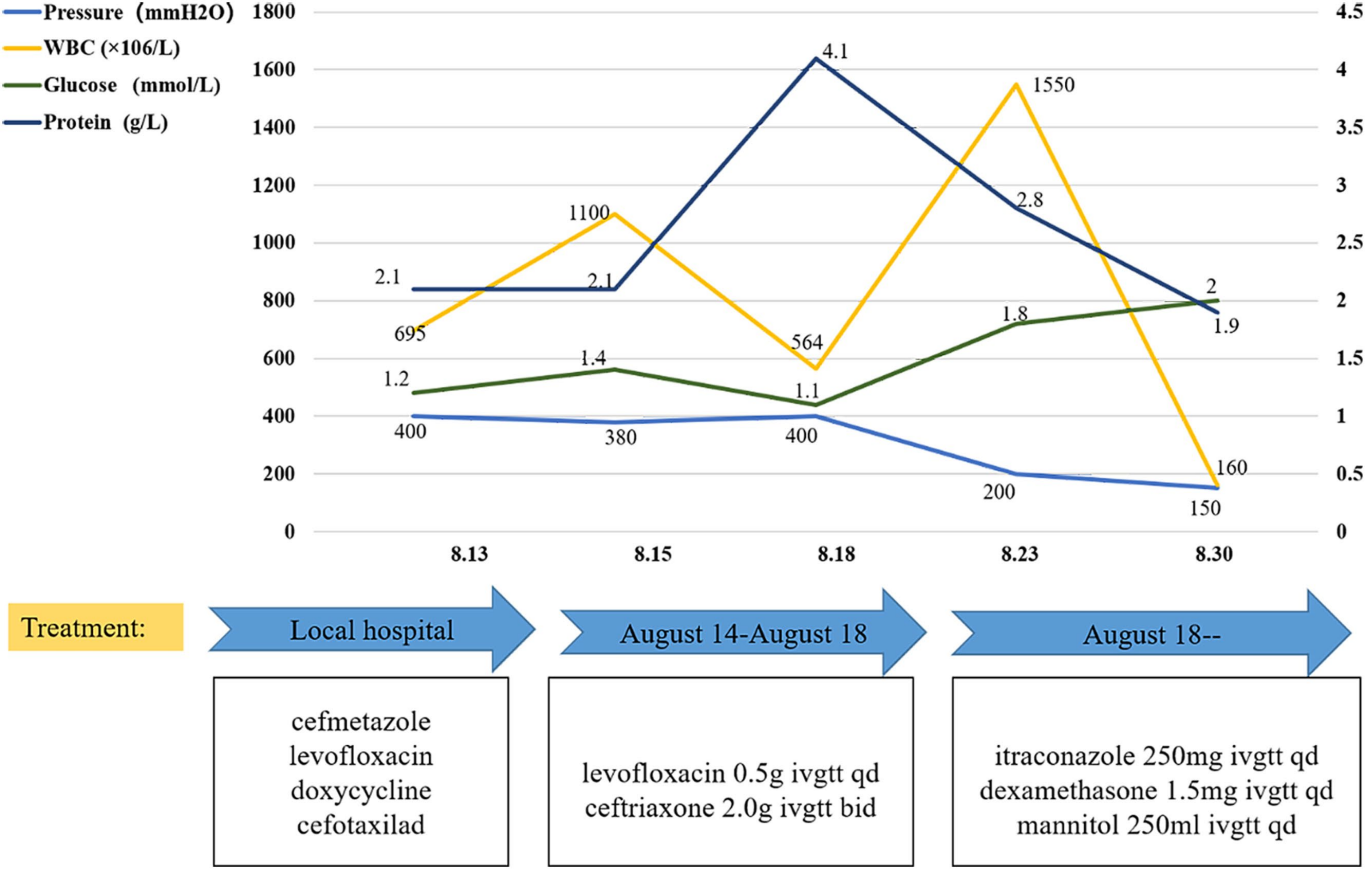
CSF investigation and antimicrobial drugs. After adjusting the treatment, the CSF factors, including glucose content, protein content, WBC, and intracranial pressure, gradually returned to normal.

Upon admission, the chest CT scan showed scattered inflammation in the lower lobe of the left lung, but no abnormalities were observed in the head during the enhancement CT scan (Figure 1B). Except for the routine blood test showing increased leukocytosis and eosinophilia, all other laboratory exams, including urinalysis, blood routine, liver and kidney function, blood glucose, blood ketones, coagulation function, procalcitonin, and myocardial markers, showed no abnormalities. As headache was the most critical sign, lumbar puncture was performed on 13 August. The CSF analysis revealed positive Pandy test (protein 2,071 mg/L), intracranial pressure greater than 300 mmH_2_O, a white blood cell count (695 × 10^6^/L) higher than the normal range, and glucose (1.2 mmol/L) and chloride(115 mmol/L) levels lower than the normal range (Figure 2). All the above results indicated the possibility of an infection in the central nervous system. Empiric combinatory therapy (levofloxacin 0.5 g ivgtt qd and ceftriaxone 2.0 g ivgtt bid) for bacterial meningitis was administered. However, there was no relief from the symptoms.

As all etiological examinations showed negative results, CSF mNGS analysis was performed to further identify the pathogen. On 17 and 19 August, two CSF mNGS analyses suggested low reads of *C. immitis* (only four unique sequence reads matched). mNGS is a novel gene sequencing approach used to rapidly identify pathogens in clinical samples, but it cannot be used as a diagnosis method when only low reads of suspicious pathogens are detected (11). Due to the detection of *C. immitis*, which is generally considered to be limited to the Western Hemisphere, the patient’s travel history was further reviewed. The patient had lived in Los Angeles, USA, for approximately 3 months (March 2018 ~ June 2018) to support his wife with child-rearing. During this period, he grew scallions in his garden and had contact with the soil.

**Figure 3:**
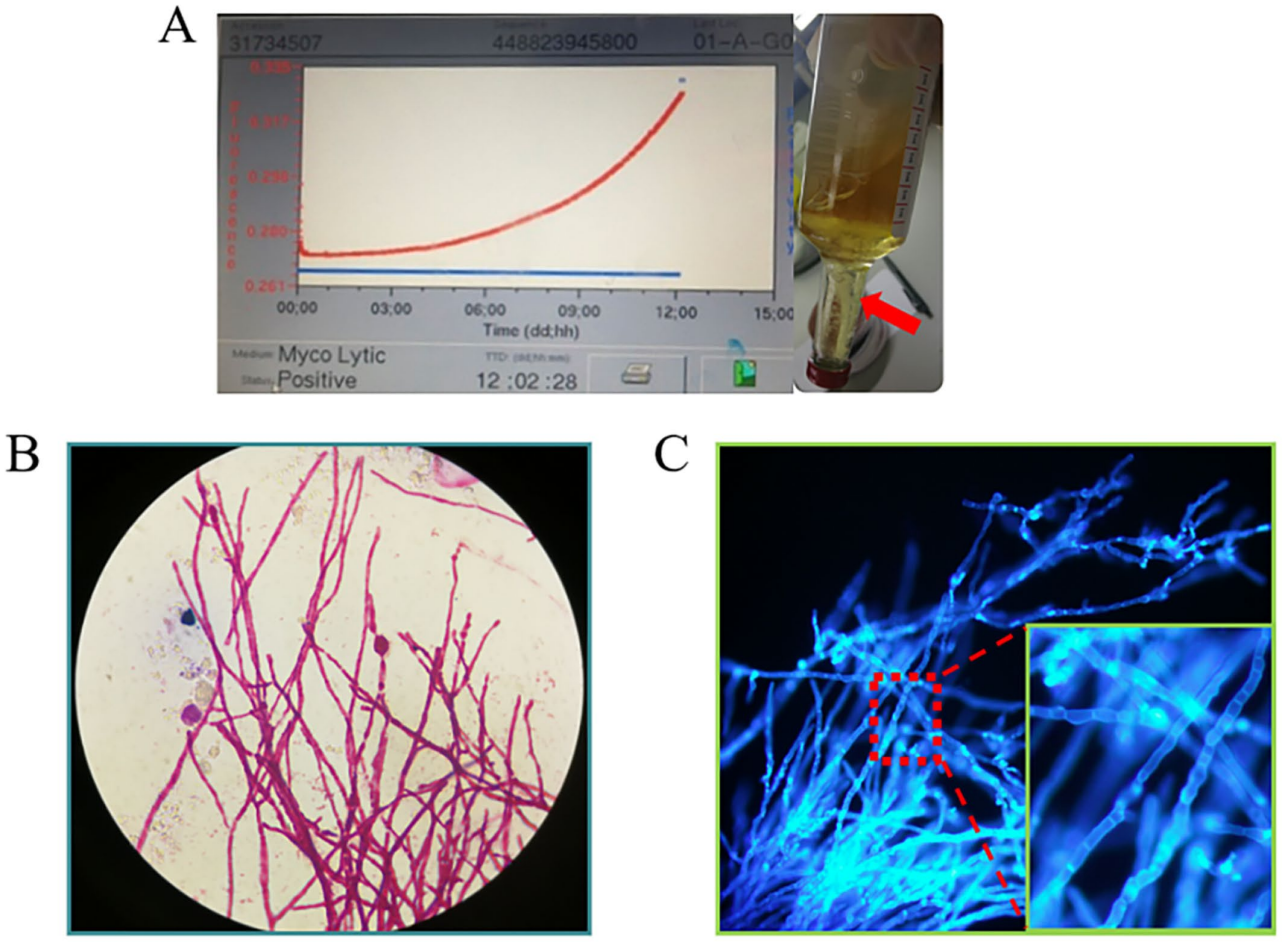
**(A)** Cerebrospinal fluid fungal culture flask annulation. After 12 days of continuous monitoring, the flask reported positivity (with an increasing growth curve). Cotton wool-like precipitation (indicated by red arrow) was visible in the bottle; **(B,C)** The cottony material was extracted and subjected to Gram staining and fluorescent staining separately. At high magnification (10×40), thick-walled spheroids and segmented hyphae were visible.

**Figure 4:**
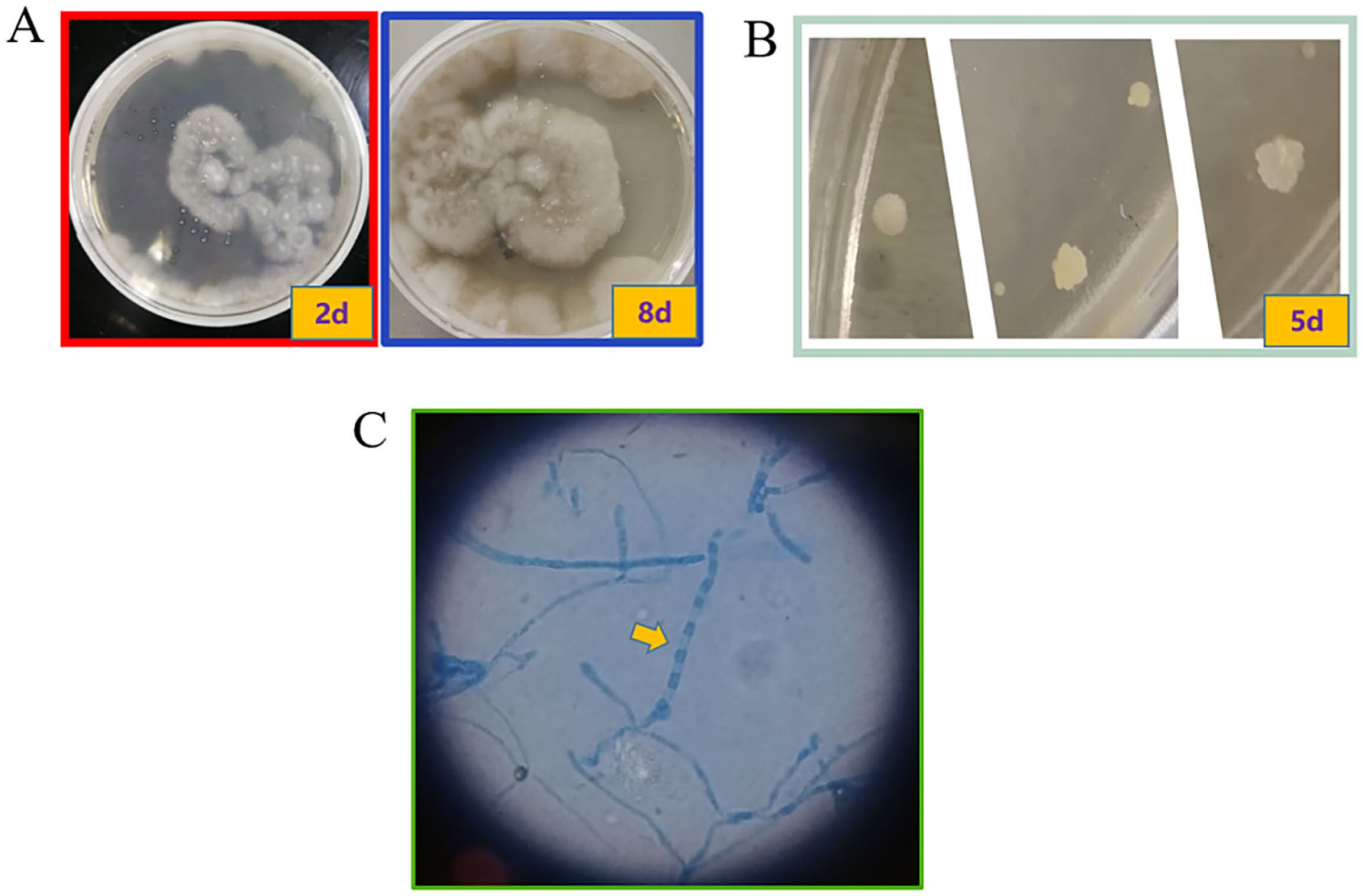
Colony on SDA plates after incubation at different temperatures. *Coccidioides* is a temperature-dependent dimorphic fungus that exhibits different colony morphologies at different temperatures. **(A)** The colony grown at 28°C had suede to villi, gray-white, and tan to brown; **(B)** The colony grown at 35°C was in the white yeast phase. **(C)** Through lactophenol cotton blue staining, arthroconidia and a large number of rectangular, barrel-shaped arthroconidia with septa were observed. These are thin-walled, connected cells. The arthroconidia and thin-walled connected cells alternate to form chains (10×100).

The subsequent second cerebrospinal fluid mNGS test also detected *C. immitis*. To verify the mNGS results, we improved the CSF culture condition according to references (7). A cerebrospinal fluid fungal culture was then conducted. Fortunately, 12 days after sampling (August 23), the fungus/mycobacteria culture flask showed positive results, and a cotton wool-like substance was produced in the flask (Figure 3A). Then, smear staining was performed, as shown in Figure 3, and thick-walled spheroids and segmented hyphae were observed using both Gram staining and Fluorescence staining. The culture material was stained with lactophenol cotton blue, revealing the characteristic microscopic features of *Coccidioides*, including barrel-shaped arthroconidia connected by thin-walled, empty-connected cells. The Gram staining and fluorescence staining revealed the presence of branching, septate hyphae, rather than thick-walled spherules, indicating a differential diagnosis of *Malbranchea* spp. At the same time, the products were transferred to Sabouraud dextrose agar (SDA) plates. The hyphal stage was observable on the SDA plates at 28°C, whereas the yeast phase appeared at 35°C, consistent with the traits of biphasic fungi (Figures 4A,B). Articular spores, a critical characteristic of *C. immitis*, were clearly identified through colony Medan staining (Figure 4C). Based on the growth and morphological characteristics of this fungus, we confirmed the mNGS results, identifying it as *C. immitis*. Subsequently, colony ITS sequencing and homology analysis were performed using BLASTp against the NCBI database to further confirm the species. Due to the lack of access to a laboratory capable of conducting serological testing, it was not performed in this study.

Therefore, the clinic changed his treatment from antibacterial to antifungal therapy. As the first-line drug for *Coccidioides*, Itraconazole was administered according to our identification results (12, 13). The detailed treatments are shown in Figure 2. With anti-coccidioidal treatment, the patient showed gradual improvement, which further corroborated that the patient’s symptoms were indeed caused by disseminated *Coccidioides* infection. By 30 August, there was a notable improvement in the patient’s CSF compared to the previous measurements and the symptoms also significantly improved. Subsequently, the patient was followed up at the infectious disease department of our hospital. After the diagnosis, the patient was treated with itraconazole, starting with a loading dose of 200 mg IV q12h for 2 days, followed by a maintenance dose of 200 mg IV qd. After 6 months, the treatment plan was changed from IV to oral administration: itraconazole oral solution 200 mg po q12h and prednisone 5 mg BID for treatment. The patient’s condition remained stable, and he continued to be followed up in the infectious disease department. For patients with *Coccidioides* meningitis, lifelong treatment with triazole medications is recommended.

## Discussion

As a result of population mobility, coccidioidomycosis cases are increasing in China (14). In addition, coccidioidal meningitis has a high mortality rate (90% within 1 year and 100% within 2 years). Therefore, early identification of the pathogen and prompt antifungal treatment are critical for a favorable prognosis. However, the etiology examination of coccidioidal meningitis remains a clinical challenge in non-endemic areas.

The diagnosis of coccidioidomycosis is mainly based on clinical manifestations, exposure history, and laboratory tests (15). In terms of laboratory tests, mNGS has emerged as one of the routine methods for detecting unknown pathogens in clinical microbiology laboratories, with the advantages of high sensitivity and specificity (15). mNGS is particularly recommended for diagnosing infections caused by new, rare, difficult-to-culture, or mixed pathogens (11). However, the cost of mNGS sequencing is high, and it cannot directly correlate suspected pathogens with clinical symptoms. Therefore, it is important to isolate pathogens using traditional cultural methods.

In this case, both the symptoms and the regular CSF analysis suggested an infection in the CNS; however, the standard CSF cultures showed negative results (no pathogen). This may be related to the fact that the culture was only observed for 3 days at 35°C on common bacterial media, which are not suitable for the growth of *Coccidioides* (which requires 28°C, using SDA, and can take 2–9 days for growth) (8). After adjusting the cultural conditions, we successfully observed the *Coccidioides* colony and its critical morphology. This finding highlights the need for microbiology laboratories to maintain close communication with clinics. When patients report a history of travel to endemic areas and exhibit symptoms such as headache, fever, and vomiting, along with CSF analysis revealing elevated opening pressure, increased protein levels, hypoglycorrhachia, and pleocytosis (16), we should consider changing the standard culture conditions to those suitable for the growth of *Coccidioides*.

This case highlights the potential of combining mNGS with traditional cultural methods to diagnose unknown pathogens quickly and accurately.

## Data Availability

The original contributions presented in the study are included in the article/supplementary material, further inquiries can be directed to the corresponding author.

## References

[1] Weida L Zhao LG. Current situation of coccidioidomycosis in China. Chin J Mycol. (2020) 15:321–4. doi: 10.3969/j.issn.1673-3827.2020.06.001

[2] Nguyen C Barker BM Hoover S Nix DE Ampel NM Frelinger JA. Recent advances in our understanding of the environmental, epidemiological, immunological, and clinical dimensions of coccidioidomycosis. Clin Microbiol Rev. (2013) 26:505–25. doi: 10.1128/cmr.00005-13, PMID: 23824371 PMC3719491

[3] Welsh O Vera-Cabrera L Rendon A Gonzalez G Bonifaz A. Coccidioidomycosis. Clin Dermatol. (2012) 30:573–91. doi: 10.1016/j.clindermatol.2012.01.003, PMID: 23068145

[4] Liang G Shen Y Lv G Zheng H Mei H Zheng X. Coccidioidomycosis: imported and possible domestic cases in China: a case report and review, 1958-2017. Mycoses. (2018) 61:506–13. doi: 10.1111/myc.12750, PMID: 29383771

[5] Yang X Song Y Liang T Wang Q Li R Liu W. Application of laser capture microdissection and PCR sequencing in the diagnosis of Coccidioides spp. infection: a case report and literature review in China. Emerg Microbes Infect. (2021) 10:331–41. doi: 10.1080/22221751.2021.1889931, PMID: 33576325 PMC7919914

[6] Ekeng BE Davies AA Osaigbovo II Warris A Oladele RO Denning DW. Pulmonary and extrapulmonary manifestations of fungal infections misdiagnosed as tuberculosis: the need for prompt diagnosis and management. J Fungi (Basel). (2022) 8:460. doi: 10.3390/jof8050460, PMID: 35628715 PMC9143176

[7] Kassis C Durkin M Holbrook E Myers R Wheat L. Advances in diagnosis of progressive pulmonary and disseminated Coccidioidomycosis. Clin Infect Dis. (2021) 72:968–75. doi: 10.1093/cid/ciaa188, PMID: 32108231 PMC7958817

[8] Saubolle MA. Laboratory aspects in the diagnosis of coccidioidomycosis. Ann N Y Acad Sci. (2007) 1111:301–14. doi: 10.1196/annals.1406.049, PMID: 17363434

[9] Bays DJ Thompson GR 3rd. Coccidioidomycosis. Infect Dis Clin N Am. (2021) 35:453–69. doi: 10.1016/j.idc.2021.03.010, PMID: 34016286

[10] McHardy IH Barker B Thompson GR 3rd. Review of clinical and laboratory diagnostics for Coccidioidomycosis. J Clin Microbiol. (2023) 61:e0158122. doi: 10.1128/jcm.01581-22, PMID: 36883820 PMC10204634

[11] Yue L Yangyang H Xing Z Yadong W Caiyan Z. Interpretation of expert consensus on clinical application of Chinese metagenomics second-generation sequencing technology to detect infectious pathogens. J Hebei Med Univ. (2021) 42:745–9. doi: 10.3969/j.issn.1007-3205.2021.07.001

[12] Galgiani JN Ampel NM Blair JE Catanzaro A Johnson RH Stevens DA. Coccidioidomycosis. Clin Infect Dis. (2005) 41:1217–23. doi: 10.1086/496991, PMID: 16206093

[13] Ramani R Chaturvedi V. Antifungal susceptibility profiles of Coccidioides immitis and Coccidioides posadasii from endemic and non-endemic areas. Mycopathologia. (2007) 163:315–9. doi: 10.1007/s11046-007-9018-7, PMID: 17484074

[14] Stevens DA Zhang Y Finkelman MA Pappagianis D Clemons KV Martinez M. Cerebrospinal fluid (1,3)-Beta-d-glucan testing is useful in diagnosis of Coccidioidal meningitis. J Clin Microbiol. (2016) 54:2707–10. doi: 10.1128/jcm.01224-16, PMID: 27558179 PMC5078547

[15] Williams SL Chiller T. Update on the epidemiology, diagnosis, and treatment of Coccidioidomycosis. J Fungi (Basel). (2022) 8:666. doi: 10.3390/jof8070666, PMID: 35887423 PMC9316141

[16] Guo X Ruan Q Jin J Zheng J Shao L Li N. Disseminated coccidioidomycosis in immunocompetent patients in non-endemic areas: a case series and literature review. Eur J Clin Microbiol Infect Dis. (2022) 41:925–39. doi: 10.1007/s10096-022-04447-y, PMID: 35546215

